# Supplementary material for: A polyphasic approach in the identification and biochemical characterization of Dunaliella tertiolecta with biodiesel potential from a saltern in Mauritius
Source: PeerJ. 2024 Dec 12;12:e18325. doi: 10.7717/peerj.18325 (PMC11646422; doi:10.7717/peerj.18325)
Supplement: Supplemental Information 1 [file peerj-12-18325-s001.docx]

**Supplementary material**

Supplementary Table S1: Components of the modified Johnson growth medium

| **Components** | **mgL^-1^** |
| --- | --- |
| KNO_3_ | 1000 |
| KH_2_PO_4_ | 35 |
| H_3_BO_3_ | 0.61 |
| CoCl_2_ 6H_2_O | 0.051 |
| CuSO_4_ 5H_2_O | 0.060 |
| MnCl_2_ 4H_2_O | 0.041 |
| (NH_4_)_6_Mo_7_O_24_ 4H_2_O | 0.38 |
| ZnCl_2_ | 0.041 |
| FeCl_3_ 6H_2_O | 2.44 |
| Na_2_EDTA 2H_2_O | 1.89 |
| MgCl_2_ 6H_2_O | 1500 |
| MgSO_4_ 7H_2_O | 500 |
| KCl | 20 |
| CaCl_2_ 6H_2_O | 20 |
| NaHCO_3_ | 43 |
| Distilled water | 1 L |
| pH | 7.5 |

Supplementary Table S2: List of primers used in this study

| **Target region** | **Types of primer** | **Primers** | **Primer sequence (5´ to 3´)** | **References** |
| --- | --- | --- | --- | --- |
| 18S rDNA | Conserved | MA1 (f) | CGGGATCCGTAGTCATATGCTTGTCTC | (Olmos Soto, 2015) |
|  |  | MA2 (r) | CGGAATTCCTTCTGCAGGTTCACC |  |
|  |  | MA3 (r) | GGAATTCCGGAAACCTTGTTACGAC |  |
|  |  |  |  |  |
|  | Species-specific | DBs (f) | GGGAGTCTTTTTCCACCT |  |
|  |  | DPs (f) | GTAGAGGGTAGGAGAAGT |  |
|  |  | DSs (f) | GCAGGAGAGCTAATAGGA |  |
|  |  |  |  |  |
| ITS | Universal | AB1 (f) | AATCTATCAATAACCACACCG | (Hejazi et al., 2010) |
|  |  | AB2 (r) | TTTCATTCGCCATTACTAAGG |  |

(f) – forward; (r) – reverse

Supplementary Table S3: The physicochemical characteristics of water samples collected from Mauritian saltpan

| **Parameters** |  | **Estimated values** |
| --- | --- | --- |
| Salinity (g L^-1^) |  | 262 ± 7.9 |
| Temperature (°C) |  | 33.7 ± 0 |
| pH |  | 7.6 ± 0.03 |
| Electrical Conductivity (mS cm^-1^) |  | 152.52 ± 13.67 |
| Dissolved oxygen (mg L^-1^) |  | 2.25 ± 0.03 |
| **Major cations and anions (g L^-1^)** | |  |
| Na^+^ |  | 95.14 ± 9.48 |
| Ca^+^ |  | 1.36 ± 0.08 |
| Mg^2+^ |  | 13.28 ± 1.06 |
| Cl^-^ |  | 138.50 ± 5.55 |
| K^+^ |  | 4.27 ± 0.57 |
| SO_4_^2-^ |  | 17.69 ±0.8 |


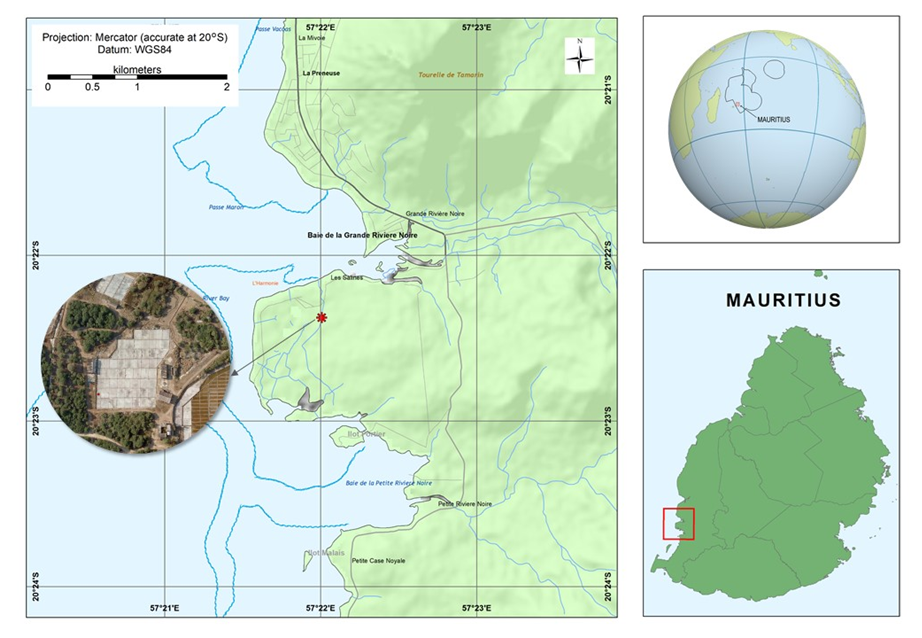


Supplementary Figure S1: Geographical map of the sampling location. The red asterisk denotes the sampling site where the water samples were collected.


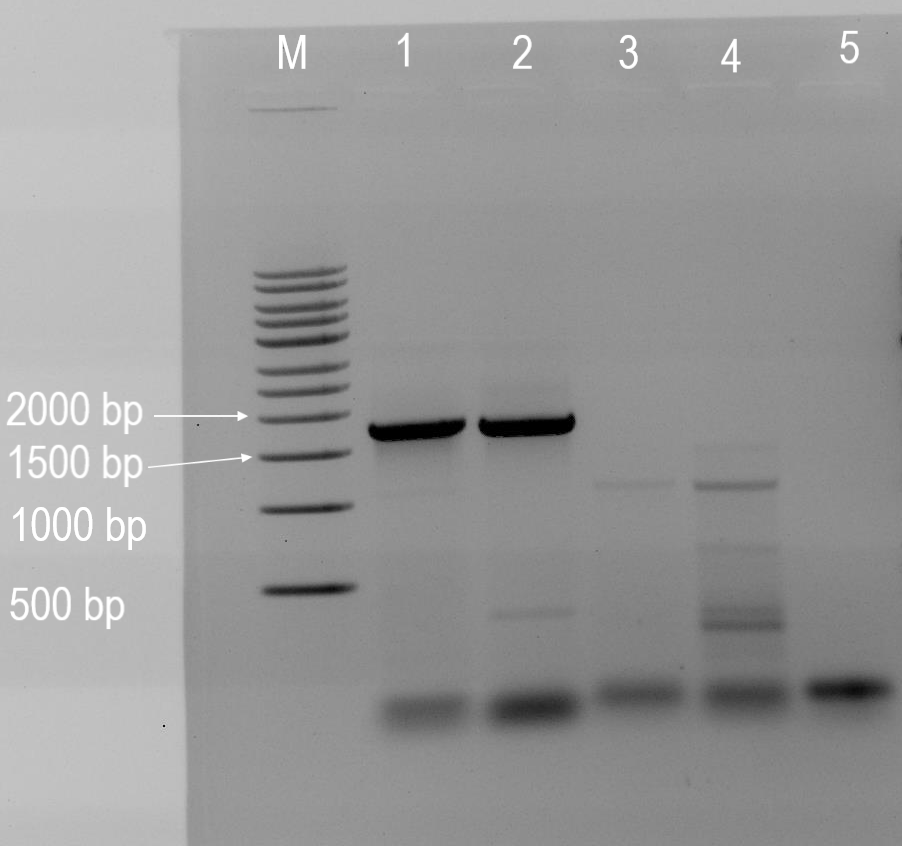


Supplementary Figure S2*:* Agarose gel electrophoresis of the PCR product of 18S rDNA gene from *Dunaliella* strain SCH18. Lane M is the 1 kb DNA ladder; Lane 1 and lane 2 correspond to amplification with MA-MA2 and MA1-MA3 primers, respectively. Lanes 3, 4, and 5 correspond to amplification with DSs-MA2, DBs-MA2, and DPs-MA
